# The chain-mediating roles of generative AI literacy and TPACK in the relationship between AI support and teaching practice among university teachers

**DOI:** 10.3389/fpsyg.2026.1932185

**Published:** 2026-07-30

**Authors:** Zhiyuan Yang, Ya’nan Lan

**Affiliations:** Faculty of International Education, Qingdao Hengxing University of Science and Technology, Qingdao, Shandong, China

**Keywords:** AI support, generative AI literacy, higher education, teaching practice, TPACK

## Abstract

**Background:**

The rapid development of generative artificial intelligence (AI) is reshaping the higher education ecosystem, and the application of AI in teaching is becoming increasingly widespread. However, many teachers still use AI primarily to improve work efficiency rather than integrating it meaningfully into teaching. This study investigated the relationships among AI support, generative AI literacy, TPACK, and teaching practice among university teachers in Shandong Province, China.

**Methods:**

A structured questionnaire was administered online via Wenjuanxing, yielding 578 valid responses. Data were analyzed using descriptive statistics, Pearson correlation analysis, confirmatory factor analysis (CFA), and structural equation modeling (SEM).

**Results:**

The results showed that all variables were significantly and positively correlated. AI support had a significant positive effect on both generative AI literacy and teaching practice. Generative AI literacy had a significant positive effect on teachers’ TPACK, and TPACK had a significant positive effect on teaching practice. Moreover, generative AI literacy and TPACK played significant chain-mediating roles in the relationship between AI support and teaching practice.

**Conclusion:**

The findings extend the application of the TPACK framework in AI-enhanced educational contexts and provide empirical evidence for higher education administrators and teachers to strengthen teachers’ generative AI literacy and improve technology-integrated teaching practices.

## 1 Introduction

The widespread adoption of generative artificial intelligence (AI) in higher education has created new opportunities for technology-enhanced teaching while also posing new challenges for teachers’ professional competence (Lin et al., 2025; Yao, 2025). In the field of education, generative AI (GAI) is developing quickly in information processing, learning ability, and problem-solving, making it a new trend in technology-empowered education (Sun and Zhou, 2024). Generative AI promotes innovation in teaching and personalized learning (Zawacki-Richter et al., 2019; Kasneci et al., 2023; Chen et al., 2024). However, relatively little research has examined whether teachers possess the competencies required to integrate generative AI effectively into pedagogy and subject-specific teaching.

In recent years, many universities have provided teachers with various AI support, including teaching platforms, training, and various AI resources (Fan et al., 2026; Tan et al., 2024). Although most teachers have applied AI in the teaching process, they generally only see it as a tool to improve work efficiency (Zeng et al., 2021; Zhang, 2025; Tripathi et al., 2025; Sari and Dwikurnaningsih, 2025; Wijanarko et al., 2025), and have not meaningfully integrated AI into teaching practices. Therefore, the process of effectively incorporating AI into teaching methods and subject teaching is still unclear.

In China, artificial intelligence is being rapidly integrated into higher education. The state encourages universities to embed AI into curriculum design, classroom instruction, and the full spectrum of teachers’ professional development (Ministry of Education of the People's Republic of China, 2025). Institutions of higher education across Shandong Province have actively advanced AI-enabled teaching reforms by constructing digital infrastructure, launching specialized AI training programs, and deploying smart teaching platforms (Shandong Provincial Department of Education, 2025). Such policy initiatives have substantially accelerated the adoption of AI tools among university teachers. Nevertheless, despite continuous institutional investment in relevant resources, numerous teachers still encounter substantial obstacles when translating AI into authentic classroom teaching, primarily due to uneven levels of AI literacy and technology integration competence across the teaching workforce. Multiple empirical studies indicate that university teachers generally possess moderate-to-upper levels of AI literacy, yet prominent individual disparities persist in terms of in-depth theoretical knowledge, practical skill application, and ethical awareness. Sustained training opportunities, organizational support, and personal motivational drive constitute the core determinants of literacy improvement (Li et al., 2025; Dou, 2026).

The TPACK model is one of the most influential teacher competency models in current international educational technology research (Mishra and Koehler, 2006; Koehler et al., 2013). It emphasizes that truly effective technology integration is not just about the technology itself, but about the deep integration of technology, pedagogy, and subject content (Mishra and Koehler, 2006; Carrió, 2025). Existing research shows that the TPACK model has been widely applied in studies on technology integration in teachers’ teaching processes (Voogt et al., 2013; Cheng et al., 2025; Abdelhalim, 2026). Compared with traditional ICT, generative AI systems are interactive, provide autonomous feedback, and offer contextualized responses.

Therefore, the ability to effectively integrate AI requires a deep fusion of technology, pedagogy, and subject content.

Existing literature has identified a multitude of predictors shaping teachers’ technology-integrated teaching, such as technological self-efficacy, technology acceptance, teachers’ pedagogical beliefs and technological readiness (Ertmer and Ottenbreit-Leftwich, 2010; Runge et al., 2025). These factors adequately explain teachers’ subjective willingness, self-confidence and readiness to adopt emerging educational technologies. Nevertheless, the current study uniquely focuses on a developmental pathway that delineates how institutional external support translates into teachers’ professional competencies and further materializes into authentic classroom teaching behaviors.

From the lens of teacher professional development, the four variables—AI support, generative AI literacy, TPACK and teaching practice—correspond to successive developmental phases for educators, ranging from accessing external institutional resources and developing personal capabilities to translating such capabilities into classroom implementation. To elaborate, AI support encompasses technological infrastructure, institutional guarantees and training resources offered by universities for faculty members; generative AI literacy refers to teachers’ holistic competencies to understand, critically assess, ethically and properly deploy, and practically apply artificial intelligence; Technological Pedagogical Content Knowledge (TPACK) measures teachers’ capacity to integrate technology, pedagogical methods and disciplinary content knowledge; and teaching practice represents the tangible behavioral manifestation of such integrated competencies within real classroom settings.

As such, the four core constructs form four sequential hierarchical levels: institutional AI support, individual generative AI literacy, integrated professional teaching competency, and classroom instructional behavior. By embedding all four constructs within a unified analytical model, this research not only explores whether teachers adopt artificial intelligence in teaching, but also untangles the internal mechanism through which institutional external support evolves into substantive technology-integrated instructional practices. This study introduces AI literacy to expand the TPACK theory in AI contexts and provides empirical data tailored to Chinese university settings, broadening TPACK’s application scenarios.

The main research questions of this study include:

RQ1. Are there relationships between AI support, generative AI literacy, TPACK, and teaching practice among university teachers?

RQ2. What mediating roles do generative AI literacy and TPACK play in the relationship between AI support and university teachers’ teaching practice?

## 2 Literature review

### 2.1 Theoretical framework

This study is primarily grounded in Social Cognitive Theory (SCT) (Bandura, 1986), which explains human behavior through the interaction among environmental factors, personal factors, and behavioral outcomes. According to SCT, environmental support creates opportunities for individuals to develop knowledge and competencies, which subsequently influence their behavioral performance. This theoretical perspective has been widely applied to explain teachers’ professional development and technology integration in educational settings. In the present study, AI support represents the environmental factor by providing technological resources, institutional support, and professional training. Generative AI literacy and TPACK represent teachers’ personal competencies that enable them to understand, evaluate, and integrate AI into teaching. Teaching practice is regarded as the behavioral outcome reflecting teachers’ actual implementation of AI-supported instruction. Accordingly, this study proposes that AI support promotes university teachers’ teaching practice both directly and indirectly through the sequential development of generative AI literacy and TPACK. Although AI-oriented extensions such as AI-TPACK have recently been proposed, these models remain under development and have received relatively limited empirical validation. In contrast, the original TPACK framework remains one of the most widely validated models for explaining teachers’ technology integration competence (Mishra and Koehler, 2006; Koehler et al., 2013). Therefore, this study adopts the original TPACK framework while incorporating generative AI literacy as an AI-specific competency to explain technology-integrated teaching in the era of generative AI.

### 2.2 AI support and AI literacy

AI support encompasses ICT support, institutional support, and training support (Yang and Li, 2026). From the perspective of teacher professional development, institutional support serves as a vital external prerequisite for teachers to acquire new professional competencies. Universities that supply sufficient AI teaching resources, regular organizational guarantees, and ongoing professional training can offer teachers more opportunities to engage with and apply generative artificial intelligence technologies. Accordingly, it can be inferred that institutional AI support effectively fosters the development of teachers’ generative AI literacy.

Celik (2023) found that continuous interaction with AI tools promotes teachers’ acquisition of AI knowledge and operational skills. In addition, Guan et al. (2026) and Runge et al. (2025) reported that AI training positively influences teachers’ professional competence, AI self-efficacy, and AI readiness. Chiu (2025) found that AI support positively predicts teachers’ AI literacy and directly promotes technology-integrated teaching practices. In summary, AI support provides the essential external conditions for teachers to cultivate generative AI literacy and facilitates the practical implementation of artificial intelligence in instructional practice. Accordingly, the following hypothesis is proposed:

Hypothesis 1: AI support positively influences the generative AI literacy of university teachers.

Hypothesis 2: AI support positively influences the instructional practices of university teachers.

### 2.3 Generative AI literacy and TPACK

Generative AI literacy encompasses capabilities such as understanding AI, critically evaluating it, and interacting effectively with it (Long and Magerko, 2020). In the context of higher education, AI literacy involves mastering AI technologies, critically assessing the validity of outcomes, and adhering to ethical standards. It emphasizes that AI literacy goes beyond mere technical proficiency by focusing on ensuring that the application of AI in teaching practices is both meaningful and ethically sound (Ng et al., 2024). From the theoretical perspective of TPACK, to achieve effective integration of artificial intelligence into teaching, teachers are required to possess not only operational proficiency in AI tools but also an understanding of AI’s educational value, applicable boundaries, and ethical norms. Generative AI literacy equips teachers with the foundational knowledge and critical competencies necessary to comprehend, evaluate, and deploy artificial intelligence, enabling them to identify AI-generated information, select AI tools suitable for instructional scenarios, and utilize AI responsibly within educational contexts. Such comprehensive capabilities allow teachers to transform isolated technical knowledge into integrated pedagogical knowledge, thereby advancing the development of their TPACK competencies. Existing research has also demonstrated the significant role of AI literacy in education. For instance, Sperling et al. (2024) demonstrated that AI literacy positively predicts teachers’ instructional decision-making capability. Daher (2025) reported a significant positive relationship between AI literacy and classroom teaching quality. Deshen et al. (2026) found that AI literacy significantly promotes teachers’ ethical use of AI technologies. Accordingly, the following hypothesis is proposed:

Hypothesis 3: Generative AI literacy positively influences the TPACK capabilities of university teachers.

### 2.4 TPACK and teaching practice

TPACK was developed by Mishra and Koehler (2006) based on the concept of Pedagogical Content Knowledge (PCK). This theory emphasizes the integration of technological knowledge, pedagogical knowledge, and content knowledge to form a highly adaptive, comprehensive, and dynamic instructional system. The framework highlights that, rather than only applying technology in a simple manner, the key to high-quality classroom instruction lies in a teacher’s ability to fuse technology, pedagogy, and subject matter to achieve effective instructional design and classroom implementation. Furthermore, while traditional educational technologies serve as passive, auxiliary tools, generative AI is characterized by interactivity, autonomous feedback, and contextualized responses. Consequently, effective AI-supported teaching requires teachers not only to use AI technologies but also to integrate them with pedagogy and subject content.

Existing research has consistently demonstrated that TPACK is one of the strongest predictors of teachers’ technology-integrated teaching practices (Voogt et al., 2013; Tondeur et al., 2017). From the perspective of the TPACK framework, teachers’ effective use of generative AI depends not only on their ability to operate AI tools but also on their capacity to integrate AI with pedagogical strategies and subject content. Teachers with higher levels of TPACK are more capable of selecting appropriate AI tools, designing AI-supported learning activities, adapting instructional strategies to different learning contexts, and facilitating meaningful student learning. Accordingly, teachers with stronger TPACK are expected to demonstrate higher levels of technology-integrated teaching practice.

However, Chiu (2025) and Wang et al. (2025) found that AI literacy positively contributes to the development of teachers’ TPACK, although the two constructs represent distinct competencies. Seunmin and Anna (2024) demonstrated that teachers with stronger AI literacy are better able to understand and utilize AI tools. More importantly, success depends on whether teachers can meaningfully integrate AI technology into specific instructional contexts. Tan et al. (2025) reported that AI positively influences classroom learning outcomes only when teachers effectively integrate AI with pedagogical strategies and subject content. Therefore, TPACK is regarded as a critical mechanism for AI-integrated teaching competence. Since AI support facilitates teachers’ generative AI literacy, generative AI literacy contributes to the development of TPACK, and TPACK subsequently promotes technology-integrated teaching practice, generative AI literacy and TPACK are expected to function as sequential mediators linking AI support and teaching practice. Accordingly, the following hypotheses are proposed:

Hypothesis 4: TPACK positively influences university teachers’ teaching practice.

Hypothesis 5: Generative AI literacy and TPACK sequentially mediate the relationship between AI support and university teachers’ teaching practice.

## 3 Methodology

### 3.1 Participants and sampling

This study focuses on frontline university teachers in Shandong Province. The participants were university teachers who were responsible for course design and classroom instruction. During the teaching process, they were required to integrate subject content, pedagogical approaches, and digital technologies while implementing classroom instruction. Therefore, this group was considered appropriate for examining university teachers’ teaching practices in the context of AI-supported education.

This study employed a structured questionnaire to collect data. An online questionnaire link was generated using the “Wenjuanxing” platform. The introductory page outlined the study’s purpose and completion instructions, explicitly informing participants that the survey was anonymous and involved no collection of personally identifiable information. Participation was voluntary and based on the principle of informed consent.

The questionnaire link was distributed through WeChat and QQ groups, resulting in a total of 659 returned questionnaires. After excluding questionnaires with nearly identical response patterns, excessively short completion times, or substantial missing data, 578 valid questionnaires were retained, yielding a valid response rate of 87.7%. The final sample was considered adequate for the subsequent statistical analyses.

Participating universities had generally introduced digital learning platforms, AI-related training activities to support teachers’ use of AI in teaching. However, the extent to which teachers perceived and utilized these support mechanisms varied across institutions. Therefore, this study focused on teachers’ perceived AI support rather than objective institutional conditions.

Table 1 presents the demographic characteristics of the participants. Of the 578 respondents, 62.8% were female and 37.2% were male. The largest age group was teachers younger than 40 years (42.4%), followed by those aged 40–50 years (32.9%) and 50 years or above (24.7%). Regarding teaching experience, 34.8% of the participants had 11–20 years of teaching experience, representing the largest group. In addition, 75.4% of the respondents were employed at public universities, whereas 24.6% worked at private universities. These demographic characteristics provide a clear description of the study sample and enhance the transparency of the participant information.

**Table 1:** Demographic characteristics of participants.

Characteristic	Category	*n*	%
Gender	Male	215	37.2
	Female	363	62.8
Age	<40	245	42.4
	40–50	190	32.9
	≥50	143	24.7
Teaching experience	<5 years	85	14.7
	5–10 years	164	28.4
	11–20 years	201	34.8
	>20 years	128	22.1
Institution type	Public university	436	75.4
	Private university	142	24.6

*N* = 578.

### 3.2 Research instruments

The questionnaire comprised four constructs and 39 measurement items: AI support (9 items), generative AI literacy (15 items), TPACK (12 items), and teaching practice (3 items). All items were measured using a five-point Likert scale ranging from 1 (strongly disagree) to 5 (strongly agree).

The AI support scale was developed with reference to Yang and Li (2026). The scale was adapted to the context of generative AI-supported teaching in Chinese universities and expanded into three dimensions: technical support, institutional support, and training support, resulting in a nine-item instrument.

Generative AI literacy was measured using an adapted version of the scale developed by Lee and Park (2024). The original instrument consisted of 25 items across five dimensions. The adapted instrument comprised 15 items measuring technical proficiency, critical evaluation, communication skills, creative application, and ethical competence.

The TPACK scale was adapted from Schmidt et al. (2009). The original instrument consisted of seven knowledge domains (TK, PK, CK, TPK, TCK, PCK, and TPACK). The adapted instrument comprised 12 items representing three dimensions: technological pedagogical knowledge (TPK), technological content knowledge (TCK), and integrated TPACK. These dimensions capture teachers’ ability to integrate technology, pedagogy, and content knowledge within the context of generative AI-supported teaching while maintaining a parsimonious measurement model.

Teaching practice was measured using a three-item scale developed based on the conceptualizations of technology-integrated teaching proposed by Tondeur et al. (2012) and Ertmer and Ottenbreit-Leftwich (2010).

### 3.3 Pilot study

To ensure the reliability and validity of the research instruments, the revised questionnaire was evaluated through expert review and a pilot study. The instrument was assessed in terms of content validity, internal consistency, corrected item-total correlation (CITC), and exploratory factor analysis (EFA).

The translated questionnaire first underwent expert evaluation. Specifically, two experts in education reviewed the content validity and contextual appropriateness of the measurement items. Two Chinese–English bilingual experts conducted the translation and back-translation following Brislin’s (1970) procedure, while one expert in Chinese linguistics reviewed the linguistic clarity and readability of the Chinese version. Based on the experts’ recommendations, items with overlapping meanings, limited relevance to the research objectives, or inadequate applicability to the context of generative AI-supported teaching were revised or removed before the pilot study.

Subsequently, a pilot study involving 167 university teachers was conducted using the revised questionnaire. The results showed that Cronbach’s alpha coefficients for the AI Support, Generative AI Literacy, TPACK, and Teaching Practice scales ranged from 0.798 to 0.911, while all corrected item-total correlation (CITC) values exceeded 0.30, indicating satisfactory internal consistency (see Table 2). Furthermore, the EFA results showed that the extracted factor structure was consistent with the hypothesized dimensions. The cumulative variance explained exceeded 70% for all constructs, and all factor loadings were greater than 0.50 (see Table 3). No additional items were removed following the pilot study because all reliability and validity indices met the recommended criteria. Therefore, the revised questionnaire was considered appropriate for the formal survey.

**Table 2:** Reliability analysis of the pilot study instruments (*N* = 167).

Construct	No. of items	Cronbach’s *α*	CITC range
AI support	9	0.798	0.389–0.549
AI literacy	15	0.865	0.407–0.643
TPACK	12	0.911	0.554–0.714
Teaching practice	3	0.881	0.736–0.790

**Table 3:** Exploratory factor analysis results of the pilot study instruments (*N* = 167).

Construct	KMO	Bartlett’s *χ*^2^(df)	*p*	Variance explained (%)	Number of factors
AI support	0.729	629.291 (36)	<0.001	73.80	3
AI literacy	0.796	1774.765 (105)	<0.001	74.12	5
TPACK	0.879	1552.428 (66)	<0.001	76.35	3
Teaching practice	0.739	268.277 (3)	<0.001	81.47	1

### 3.4 Data analysis

Data analysis for this study was conducted using SPSS 29.0 and AMOS 28.0. Specifically, the analysis included descriptive statistics, Pearson correlation analysis, and structural equation modeling. Descriptive analysis was used to calculate the mean, standard deviation, kurtosis, and skewness of the data. Pearson correlation analysis was employed to examine the correlations between variables, while structural equation modeling was used to test the hypothesized relationships among them. Additionally, exploratory factor analysis (EFA) and confirmatory factor analysis (CFA) were conducted to validate the instrument’s validity and the measurement model, respectively.

## 4 Results

### 4.1 Common method bias test

Because all data were collected using self-reported questionnaires, common method bias (CMB) was assessed using Harman’s single-factor test and the full collinearity assessment. The Harman’s single-factor test showed that the first unrotated factor accounted for 32.8% of the total variance, which was below the recommended threshold of 40%, indicating that common method bias was unlikely to be a serious concern. Furthermore, the full collinearity assessment showed that the variance inflation factor (VIF) values for all latent constructs were below the recommended threshold of 3.3 (Kock, 2015), providing further evidence that common method bias did not substantially affect the results of this study.

### 4.2 Measurement model assessment

#### 4.2.1 Reliability

Table 4 presents the reliability results for all constructs. Cronbach’s *α* values ranged from 0.814 to 0.905, exceeding the recommended threshold of 0.70, indicating satisfactory internal consistency. Similarly, the composite reliability (CR) values ranged from 0.857 to 0.904, all above the recommended criterion of 0.70, demonstrating good construct reliability. These results indicate that all measurement constructs possess adequate reliability and are appropriate for subsequent analyses (Hair et al., 2019; Fornell and Larcker, 1981).

**Table 4:** Construct reliability.

Construct	Cronbach’s *α*	CR
Teaching practice	0.905	0.857
AI support	0.814	0.901
AI literacy	0.884	0.858
TPACK	0.888	0.904

#### 4.2.2 Confirmatory factor analysis (CFA)

Table 5 shows the fit results of the measurement model. The CFA results indicate that all the fit indices meet the recommended standards: *χ*^2^/df = 2.220, which is less than the standard value of 3; CFI = 0.973, TLI = 0.966, IFI = 0.973, all above the standard value of 0.90; RMSEA = 0.045 and SRMR = 0.042, both below the standard value of 0.08. These results indicate that the proposed measurement model demonstrated a good fit to the observed data. Because the initial CFA model achieved satisfactory fit indices, no item deletion or model modification was required during the CFA procedure.

**Table 5:** Measurement model fit indices.

Fit index	Value	Recommended value
*χ*^2^/df (CMIN/DF)	2.220	<3.0
SRMR	0.042	<0.08
CFI	0.973	>0.90
TLI	0.966	>0.90
IFI	0.973	>0.90
RMSEA	0.045	<0.08

#### 4.2.3 Convergent validity

Table 6 presents the standardized factor loadings and average variance extracted (AVE) values used to assess convergent validity. The AVE values ranged from 0.647 to 0.758, exceeding the recommended threshold of 0.50 (Fornell and Larcker, 1981). The standardized factor loadings ranged from 0.754 to 0.903, all exceeding the recommended threshold of 0.70 (Hair et al., 2019) and were statistically significant (*p* < 0.001). Therefore, all retained items were preserved for subsequent analyses because they demonstrated satisfactory convergent validity. Overall, the measurement model exhibited good convergent validity (Fornell and Larcker, 1981; Hair et al., 2019).

**Table 6:** Convergent and discriminant validity.

Construct	Factor loadings	(AVE)	1	2	3	4
Teaching practice	0.824–0.903	0.667	0.817	0.525	0.630	0.627
AI support	0.803–0.834	0.647	0.525	0.804	0.464	0.528
AI literacy	0.784–0.823	0.669	0.630	0.464	0.818	0.637
TPACK	0.754–0.852	0.758	0.627	0.528	0.637	0.870

#### 4.2.4 Discriminant validity

Table 6 also presents the results of the discriminant validity assessment. The square root of the AVE for each construct exceeded its correlations with all other constructs, satisfying the Fornell–Larcker criterion. These findings indicate that the four latent constructs demonstrate satisfactory discriminant validity (Fornell and Larcker, 1981; Hair et al., 2019), confirming that the study constructs are empirically distinct from one another.

### 4.3 Descriptive statistics and correlation analysis

Table 7 presents the descriptive statistics and Pearson correlation coefficients among the study variables. The mean scores ranged from 3.69 to 3.79, with standard deviations ranging from 0.80 to 1.15. The skewness values ranged from −0.804 to −0.526, and the kurtosis values ranged from −0.547 to −0.096, indicating that all variables approximated a normal distribution, as the values fell within the recommended range of ±2 (Hair et al., 2019).

**Table 7:** Descriptive statistics and correlations.

Variable	Mean	SD	Skewness	Kurtosis	1	2	3	4
TP	3.79	1.15	−0.758	−0.547				
AIS	3.69	0.84	−0.545	−0.393	0.525**			
AIL	3.69	0.80	−0.526	−0.302	0.630**	0.464**		
TPACK	3.79	0.94	−0.804	−0.096	0.627**	0.528**	0.637**	

TP, teaching practice; AIS, AI support; AIL, AI literacy.

**p < 0.01.

The correlation analysis results show that all variables are significantly positively correlated (*p* < 0.01). Specifically, AI support is significantly positively correlated with generative AI literacy (*r* = 0.464, *p* < 0.01), TPACK (*r* = 0.528, *p* < 0.01), and teaching practice (*r* = 0.525, *p* < 0.01); generative AI literacy is significantly positively correlated with TPACK (*r* = 0.637, *p* < 0.01) and teaching practice (*r* = 0.630, *p* < 0.01); and there is also a significant positive correlation between TPACK and teaching practice (*r* = 0.627, *p* < 0.01). These findings indicate significant associations among the study variables and provide an empirical basis for the subsequent structural equation modeling analysis.

### 4.4 Structural model assessment

Table 8 reports the fit indices of the structural model. The results show that all the fit indices of the structural model meet the recommended standards. Specifically, the chi-square to degrees of freedom ratio (*χ*^2^/df = 2.342) is less than 3.0; the comparative fit index (CFI = 0.970), Tucker–Lewis index (TLI = 0.962), and incremental fit index (IFI = 0.970) are all above 0.90; the root mean square error of approximation (RMSEA = 0.047) is below 0.08. These results indicate that the structural model fits the sample data well, allowing for further path analysis and hypothesis testing.

**Table 8:** Structural model fit indices.

Fit index	Value	Recommended value
*χ*^2^/df (CMIN/DF)	2.342	<3.0
CFI	0.970	>0.90
TLI	0.962	>0.90
IFI	0.970	>0.90
RMSEA	0.047	<0.08

**Figure 1:**
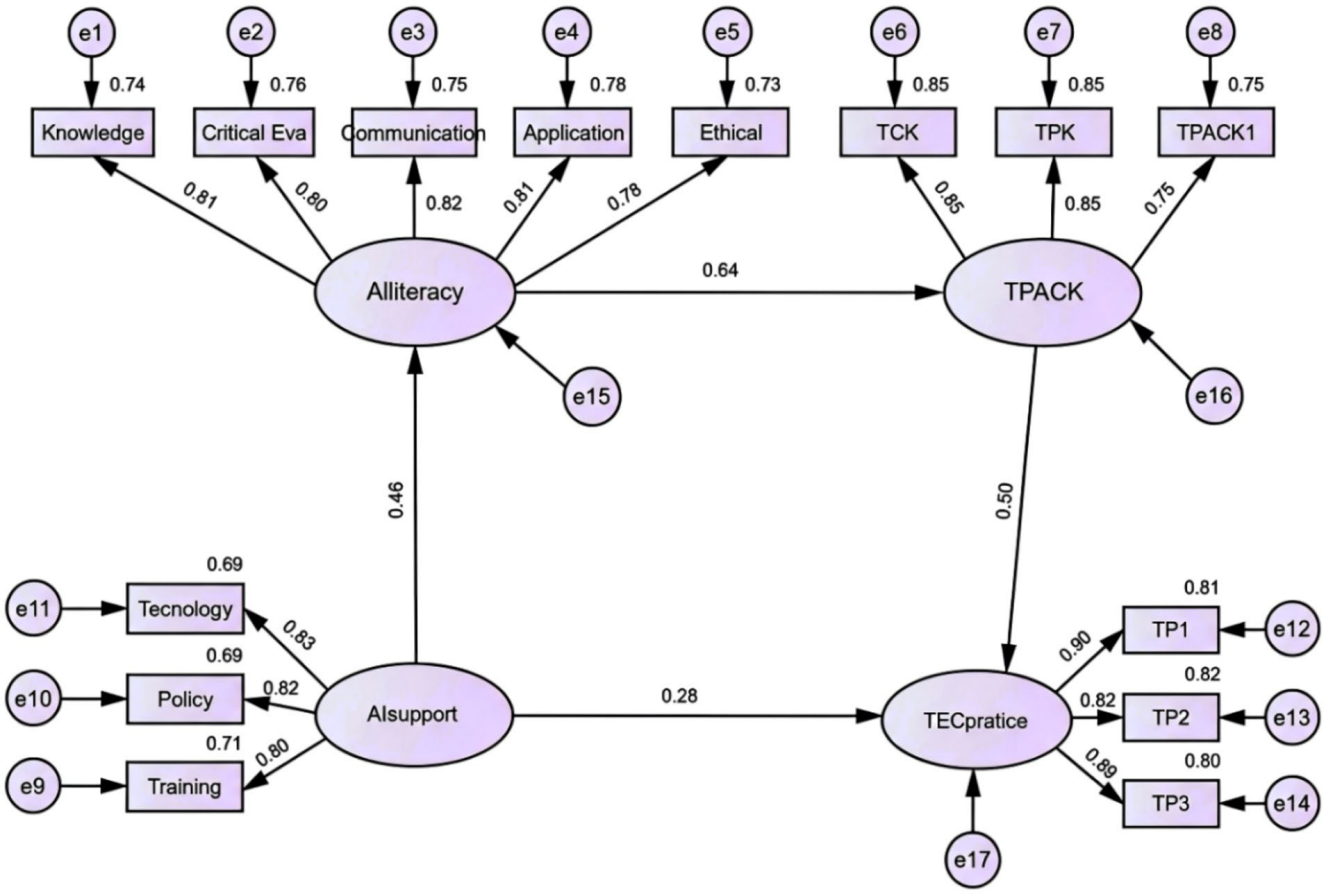
Structural model and hypothesized relationships.

Figure 1 shows the final structural model and the hypothesized relationships among the research variables.

### 4.5 Hypothesis testing

Table 9 presents the results of the structural path analysis. AI support had a significant positive effect on generative AI literacy (*β* = 0.46, *p* < 0.001), supporting H1. AI support also had a significant positive effect on teaching practice (*β* = 0.28, *p* < 0.001), supporting H2. Furthermore, generative AI literacy had a significant positive effect on TPACK (*β* = 0.64, *p* < 0.001), supporting H3. Finally, TPACK had a significant positive effect on teaching practice (*β* = 0.50, *p* < 0.001), supporting H4.

**Table 9:** Direct effects among variables.

Hypothesis	Path	Standardized coefficient (*β*)	*p*
H1	AI support → AI literacy	0.46	<0.001
H2	AI support → teaching practice	0.28	<0.001
H3	AI literacy → TPACK	0.64	<0.001
H4	TPACK → teaching practice	0.50	<0.001

Among the four direct paths, generative AI literacy demonstrated the strongest standardized effect on TPACK (*β* = 0.64), followed by TPACK on teaching practice (*β* = 0.50) and AI support on generative AI literacy (*β* = 0.46). By comparison, AI support showed the smallest direct effect on teaching practice (*β* = 0.28). Overall, the standardized path coefficients indicate that generative AI literacy and TPACK exhibited larger standardized effects than AI support in the proposed structural model.

### 4.6 Chain mediation analysis

Consistent with the hypothesized structural model, the mediation analysis focused on the proposed chain-mediating pathway from AI Support to Teaching Practice through Generative AI Literacy and TPACK. To examine the proposed chain-mediating effect, a bootstrap analysis with bias-corrected confidence intervals was conducted. As shown in Table 10, the chain-mediating effect of AI Support on Teaching Practice through Generative AI Literacy and TPACK was significant [*β* = 0.147, 95% BC CI (0.079, 0.257), *p* = 0.026]. Because the 95% bias-corrected confidence interval did not include zero, the proposed chain-mediating effect was supported. Therefore, Hypothesis 5 was supported.

**Table 10:** Bootstrap analysis of the chain-mediating effect.

Indirect path	Effect (*β*)	Lower bound (95% CI)	Upper bound (95% CI)	*p*
AI support → AI literacy → TPACK → teaching practice	0.147	0.079	0.257	0.026

## 5 Discussion

The first finding of this study is that AI support was positively associated with teachers’ AI literacy and teaching practices. This finding suggests that teachers who perceived higher levels of AI support also reported higher levels of AI literacy and greater engagement in teaching practices involving AI. In recent years, Shandong has continuously advanced the digitalization of education. On one hand, higher education institutions have provided diverse AI platform resources, incentive measures, and professional training, thereby facilitating teachers’ exposure to and familiarity with AI, as well as their mastery of its usage and operational standards. On the other hand, university teachers have actively responded to calls for instructional reform by integrating AI technology into various stages of teaching. The application of AI technology has provided teachers with more efficient access to instructional resources and teaching support, which may facilitate technology-integrated teaching practices. This finding is consistent with Yang and Li (2026), who reported that institutional AI support was positively associated with teachers’ digital technology competencies and innovative teaching practices. This study further extends the applicability of the concept of AI support to the context of higher education in China. This finding also supports the environmental component of Social Cognitive Theory by suggesting that institutional support is associated with teachers’ professional competencies in AI-enhanced educational settings. Therefore, higher education institutions should enhance teachers’ AI literacy and instructional practices by providing richer and more diverse AI platform resources, formulating and optimizing supportive policies and incentives, and offering precisely targeted professional training related to AI.

The second finding of this study is that generative AI literacy was positively associated with teachers’ TPACK. This suggests that AI literacy is an important foundation for teachers to develop the ability to integrate technology, pedagogy, and content knowledge. In the context and demands of digital education, universities in Shandong are continuously strengthening the application of AI technology in teaching, which places higher demands on teachers’ ability to integrate AI into instructional practice. Teachers not only need to know how to use AI tools but also need to have the ability to integrate AI into their teaching practices. This finding is consistent with the TPACK framework proposed by Mishra and Koehler (2006) and further extends its application to generative AI-enhanced educational contexts. TPACK emphasizes that teachers need to effectively integrate technological knowledge, pedagogical knowledge, and content knowledge, while AI literacy provides an important foundation for understanding and applying emerging technologies.

Furthermore, the standardized path coefficient between generative AI literacy and TPACK was larger than those of the other direct relationships, indicating a relatively strong association between the two constructs. The findings further suggest that generative AI literacy may serve as an important antecedent to TPACK by equipping teachers with the knowledge and competencies required to integrate emerging AI technologies into teaching, thereby extending the applicability of TPACK theory to AI-enhanced educational contexts.

The third finding of this study is that university teachers’ TPACK competence was positively associated with their teaching practices. This finding suggests that, in the context of the digital transformation of education, teachers with stronger TPACK competence also tended to report higher levels of technology-integrated teaching practices. This finding is consistent with previous research. Tondeur et al. (2017) identified TPACK as a key predictor of teachers’ technology-integrated teaching behaviors, reporting that higher levels of TPACK were positively associated with more effective technology-integrated teaching practices. The findings further extend the applicability of the TPACK framework to AI-enhanced educational contexts, suggesting that TPACK remains a valuable framework for understanding teachers’ technology integration in the era of generative AI. Moreover, the results provide empirical evidence for research on TPACK in the realm of AI-empowered education. Consequently, higher education institutions should prioritize the development of faculty TPACK competence by supporting the integration of AI technology, pedagogy, and subject matter content through AI-related professional development, instructional design workshops, and communities of practice.

The final finding of this study is that generative AI literacy and TPACK were identified as significant chain mediators in the association between AI support and teaching practices. This finding suggests that the positive association between AI support and teaching practices may be explained by the proposed chain mediation involving generative AI literacy and TPACK.

Previous studies have reported positive associations between AI support, teachers’ AI literacy, and teaching practices. The present findings provide additional empirical evidence supporting the proposed chain-mediating relationships among these variables within the structural model. The findings suggest that teachers who reported higher levels of AI support also tended to report stronger generative AI literacy and TPACK, which were in turn associated with higher levels of technology-integrated teaching practices. These findings enrich the theoretical understanding of teacher professional development in AI-enhanced educational contexts and provide additional empirical evidence supporting the integration of Social Cognitive Theory and the TPACK framework. Consequently, higher education institutions should adopt a synergistic development strategy—encompassing AI support infrastructure, AI literacy enhancement, TPACK development, and the optimization of teaching practices—to advance the digital and intelligent transformation of higher education.

## 6 Conclusion

Focusing on university teachers in Shandong Province, this study explored the relationships among AI support, generative AI literacy, TPACK, and teaching practices. The findings revealed that AI support was directly associated with teachers’ teaching practices and was indirectly associated with teaching practices through generative AI literacy and TPACK. On the theoretical level, this study extends the applicability of Social Cognitive Theory by demonstrating the associations between institutional AI support, teachers’ professional competencies, and teaching practices in AI-enhanced educational settings. In addition, the findings refine the application of the TPACK framework by suggesting that generative AI literacy may function as an important antecedent to teachers’ technology integration competence. On the practical level, the findings provide empirical evidence to support universities in developing strategies for strengthening teachers’ generative AI literacy and promoting technology-integrated teaching practices.

This study has several limitations. First, the sample was drawn from only one province; therefore, the generalizability of the findings to other regions remains to be verified. Second, the use of a cross-sectional survey design precludes the determination of causal relationships between the variables. Accordingly, the proposed chain-mediating relationship should be interpreted as a statistical association rather than evidence of a temporal or causal mechanism. Future longitudinal or experimental studies are needed to further examine the proposed mediation process. Third, all variables were measured using self-report questionnaires, which may have introduced common method bias and social desirability bias despite the procedural and statistical controls adopted in this study. Future research could triangulate self-reported data with classroom observations, interviews, teaching artifacts, or learning analytics to further strengthen the validity of the findings. Finally, future studies may also include a broader geographic sample and incorporate additional variables, such as AI acceptance and AI self-efficacy, to further extend the proposed model.

## Data Availability

The raw data supporting the conclusions of this article will be made available by the authors, without undue reservation.
